# Supplementary material for: A Global Screen for Assembly State Changes of the Mitotic Proteome by SEC-SWATH-MS
Source: Cell Syst. 2020 Feb 26;10(2):133–155.e6. doi: 10.1016/j.cels.2020.01.001 (PMC7042714; doi:10.1016/j.cels.2020.01.001)

O96019 | ACL6A_HUMAN | ACTL6A BAF53 BAF53A INO80K
Monomer MW [kDa]: 47.461 Monomer expected elution fraction: 47

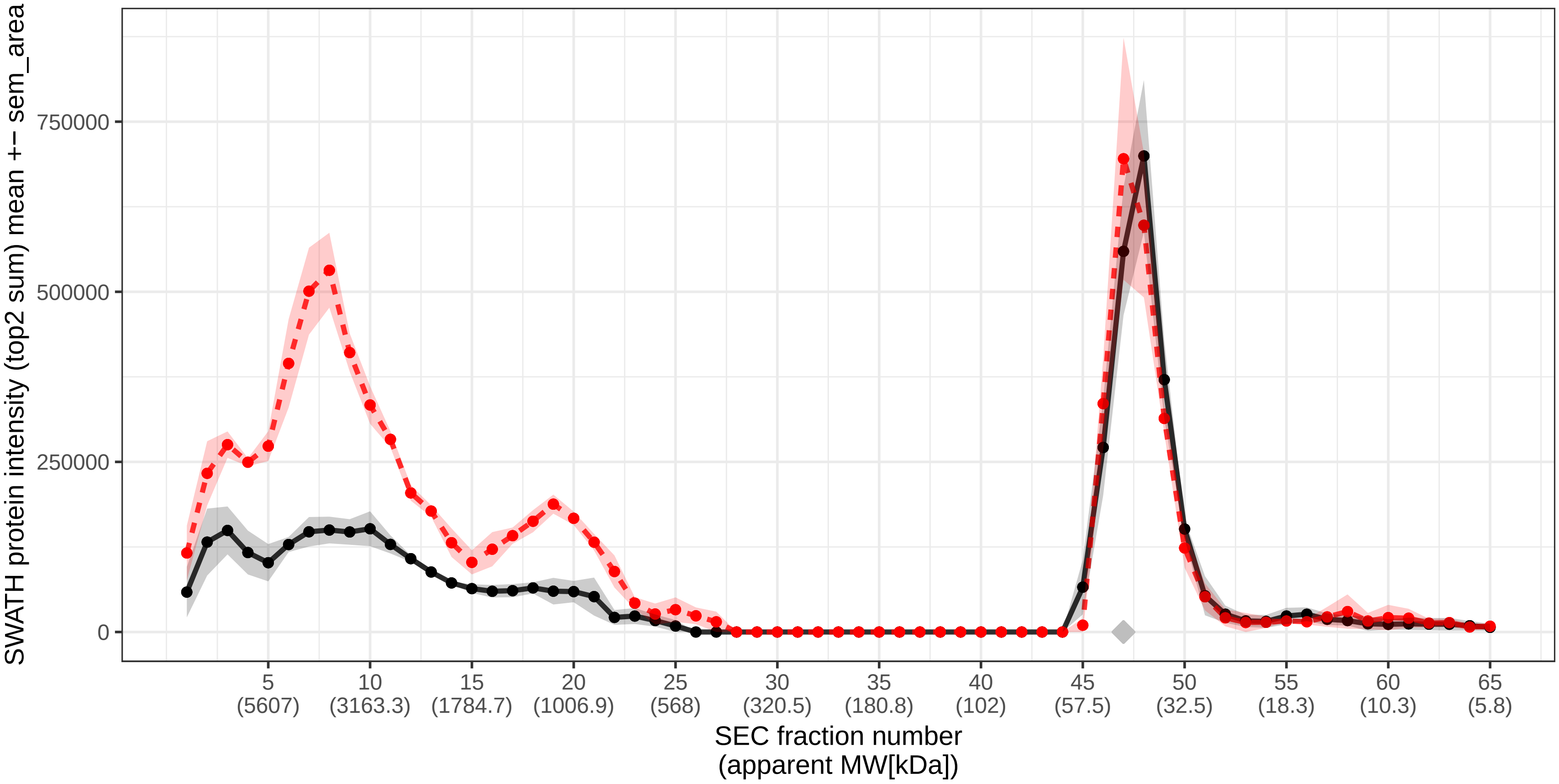

Supplement: Data S1. SEC-SWATH-MS Protein Chromatograms, Related to Figure 1 [file mmc6.zip › SECchrom_O96019_ACL6A_HUMAN_ACTL6A_BAF53_BAF53A_INO80K.pdf]
